# Structured puppy video viewing improves physiological and psychological stress responses in office workers

**DOI:** 10.3389/fpubh.2026.1726679

**Published:** 2026-07-23

**Authors:** Sang Gi Kwak, Yoon Joo Shin, Won Guk Kang, Min Young Kim, Jun Hyung Lee, Chul Park, Young Hwan Song

**Affiliations:** 1 Department of Veterinary Internal Medicine, College of Veterinary Medicine, Jeonbuk National University, Iksan, Republic of Korea; 2 Happy Dog TV Ltd., Seoul, Republic of Korea; 3 Department of Companion Animal Industry, Wonkwang University, Iksan, Republic of Korea; 4 Department of Pediatrics, Seoul National University Bundang Hospital, Seongnam, Republic of Korea

**Keywords:** animal-assisted intervention, blood pressure regulation, digital therapeutics, non-pharmacological intervention, office workers, visual stimuli, workplace stress

## Abstract

**Introduction:**

Psychological stress impairs both mental health and physiological function, making it a major occupational health risk for office workers. Although non-pharmacological emotional interventions have gained attention, scientific evidence for structured animal-based video content remains scarce. This study investigated whether structured puppy video viewing improves stress responses and compared outcomes between normal blood pressure (BP) and elevated BP office workers.

**Methods:**

Sixty-six participants (36 for normal BP, 30 for elevated BP) took part in a non-randomized pre-post intervention, viewing structured puppy videos for 15 min. Systolic BP (SBP), diastolic BP (DBP) and pulse rate were measured as physiological outcomes; psychological outcomes were assessed with the 20-item State Anxiety subscale of the State–Trait Anxiety Inventory (STAI-S) and with the 30-item the Korean Profile of Mood States-Brief (K-POMS-B), summarized as Total Mood Disturbance (TMD).

**Results:**

In normal BP group, SBP decreased by 3.16 mmHg (*p* = 0.002), pulse rate by 2.76 bpm (*p* < 0.001), STAI-S by 6.64 points (*p* < 0.001), and TMD by 7.92 points (*p* < 0.001) after viewing the video. In the elevated BP group, there were significant reductions in SBP (Δ6.65 mmHg), DBP (Δ4.93 mmHg), pulse rate (Δ4.12 bpm), and STAI-S (Δ9.43 points) (with all *p* < 0.001), alongside a TMD reduction of 11.77 points (*p* < 0.001). When comparing between normal and elevated BP groups, SBP and DBP were decreased more in the elevated BP participants (*p* < 0.049, and *p* < 0.001, respectively). Overall, structured puppy video viewing significantly alleviated both physiological and psychological stress.

**Discussion:**

These findings suggest its potential as a scalable, non-pharmacological approach to support workplace stress management.

## Introduction

In modern occupational environments, psychological stress is increasingly prevalent, underscoring the demand for accessible and non-invasive interventions that address both mental and physical health (1, 2). Psychosocial stress, including job-related stress, contributes substantially to the onset and poor management of hypertension (1, 3, 4), and may also impede BP control (3, 5–7). Psychological stress not only elevates the risk of hypertension but also impairs treatment efficacy (1) and reduces workplace productivity (8–10). As hypertension is a modifiable risk factor for cardiovascular mortality (4), addressing psychosocial contributors is critical for effective prevention and management (11, 12). This has led to growing demand for scalable, low-cost management solutions within digital health (2, 13). Beyond average BP, short-term fluctuations known as BP variability (BPV) are predictive of cardiovascular risk (14) and closely linked to psychological stress and autonomic nervous system instability (6, 7). Human-animal interaction interventions, particularly animal-assisted therapy (AAT) have attracted attention for their ability to promote positive emotional responses and reduce symptoms of anxiety and depression (15) and have also demonstrated potential to stabilize physiological parameters, including BP and pulse rate (16). However, the implementation of AAT presents practical limitations, as it depends on the use of live animals. Some individuals may harbor animal-related fears (17) or suffer from allergic reactions to pet dander (18). AAT also requires trained animals, strict adherence to hygiene and safety protocols, and careful ethical consideration of animal welfare (19, 20). These logistical and ethical challenges have led to growing interest in digital alternatives (21, 22). In line with this trend, Kogan et al. demonstrated that short animal-themed videos could enhance mood and attention in educational settings (23), suggesting broader applicability of animal video interventions beyond therapeutic contexts. Moreover, other experimental studies have shown that even brief exposure to animal video, such as puppy videos, can significantly reduce stress levels (24, 25). In addition, some studies suggest that puppy videos may be more effective than nature videos in reducing anxiety and enhancing positive emotions (25).

Earlier studies, however, primarily relied on self-report measures rather than validated psychometric instruments, and their short-term laboratory-based designs limited ecological validity (24, 25). Puppies’ faces often display baby-schema characteristics –such as a round face, large eyes, and a small nose and mouth – which can elicit emotional and physiological responses. The concept of baby schema was first introduced by Lorenz, who proposed that specific facial features promote caregiving behaviors (26). Borgi and Cirulli experimentally demonstrated that these features not only enhance perceptions of cuteness but also strengthen emotional bonding, with puppy faces eliciting the strongest response compared with dogs, cats, kittens, and even human infants (27). Building on this evidence, the present study employed puppy videos as structured emotional stimuli. In addition, Nittono et al. showed that viewing cute images promotes attentional focus and more careful behavior (28), findings consistent with earlier work by Sherman et al., who also demonstrated that exposure to cute images increases behavioral carefulness (29). Digital emotional interventions that utilize emotionally engaging content have garnered increasing interest, yet clinical evidence remains limited (30). This study evaluated the effects of scientifically structured puppy videos on physiological and psychological stress responses among Korean office workers. Accordingly, the study presents (1) whether scientifically structured puppy video viewing mitigates physiological and psychological stress indicators, and (2) whether such effects differ between the normal and elevated BP individuals.

## Materials and methods

### Participants

A total of 66 Korean office workers participated in this study (37 males, 29 females). Participants were recruited through a structured convenience sampling approach from financial and construction companies. Written informed consent was obtained from all participants prior to the study (JBNU IRB 2024–09–014-001).

Initially, 71 employees volunteered for the study. On the day of the experiment, 5 individuals were excluded due to taking of alcohol or medication within 24 h. Consequently, 66 participants were included in the final analysis. Following the 2025 ACC/AHA guidelines (31), participants were classified into two groups: the normal BP group (SBP < 120 mmHg and DBP < 80 mmHg; *n* = 36) and the elevated BP(SBP ≥ 120 mmHg and/or DBP ≥ 80 mmHg; *n* = 30) on the basis of measured BP values at the beginning of study (Table 1).

**Table 1:** Baseline characteristics of the study participants categorized by blood pressure status.

Variable	Normal BP (*n* = 36)	Elevated BP (*n* = 30)	*p*-value
Demographics and anthropometrics			
Age (years)	38.64 ± 8.34	42.07 ± 8.99	0.113
Sex (Male/Female)	14 (38.9%) / 22 (61.1%)	23 (76.7%)/7 (23.3%)	0.002
Height (cm)	167.58 ± 8.25	171.23 ± 7.78	0.071
Weight (kg)	63.14 ± 15.16	74.67 ± 11.75	0.001
BMI (kg/m^2^)	22.29 ± 3.45	25.38 ± 3.30	<0.001
Education			0.378
College graduate	3 (8.3%)	2 (6.7%)	
University graduate	25 (69.4%)	25 (83.3%)	
Graduate degree	8 (22.2%)	3 (10.0%)	
Occupational characteristics			
Functional Role			0.008
Revenue-generating	29 (80.6%)	15 (50.0%)	
Operational support	7 (19.4%)	15 (50.0%)	
Years of service (years)	7.53 ± 7.42	11.40 ± 8.40	0.051
Weekly workload (hours)	40.0 ± 0.0	40.0 ± 0.0	–
Anti-hypertension medication (Yes)	0 (0.0%)	6 (20.0%)	0.007
Cardiovascular measures (baseline)			
Systolic BP (mmHg)	104.03 ± 9.83	125.23 ± 13.53	< 0.001
Diastolic BP (mmHg)	71.29 ± 6.24	88.28 ± 7.44	< 0.001
Pulse rate (bpm)	75.49 ± 8.32	82.95 ± 11.59	0.003
Pet-related characteristics			
Pet ownership			0.934
Past	16 (44.4%)	12 (40.0%)	
Present	8 (22.2%)	7 (23.3%)	
Never	12 (33.3%)	11 (36.7%)	
Dog affinity			0.285
Positive	28 (77.8%)	18 (60.0%)	
Neutral	7 (19.4%)	10 (33.3%)	
Negative	1 (2.8%)	2 (6.7%)	
Psychological measures (baseline)			
STAI-S	43.06 ± 8.38	41.43 ± 12.91	0.541
Tension	5.39 ± 4.53	5.47 ± 4.34	0.944
Depression	4.03 ± 3.75	4.47 ± 4.57	0.670
Anger	4.72 ± 3.88	3.93 ± 3.76	0.407
Fatigue	6.58 ± 3.89	6.70 ± 4.58	0.911
Confusion	7.78 ± 3.09	6.27 ± 3.13	0.054
Vigor	10.58 ± 4.12	9.37 ± 3.82	0.221
TMD	17.97 ± 18.97	17.73 ± 20.95	0.961

Values are presented as Mean ± SD or *n* (%). BP, blood pressure; BMI, body mass index; STAI-S, State–Trait Anxiety Inventory-State; TMD, Total Mood Disturbance.

### Stimuli (video intervention)

**Figure 1:**
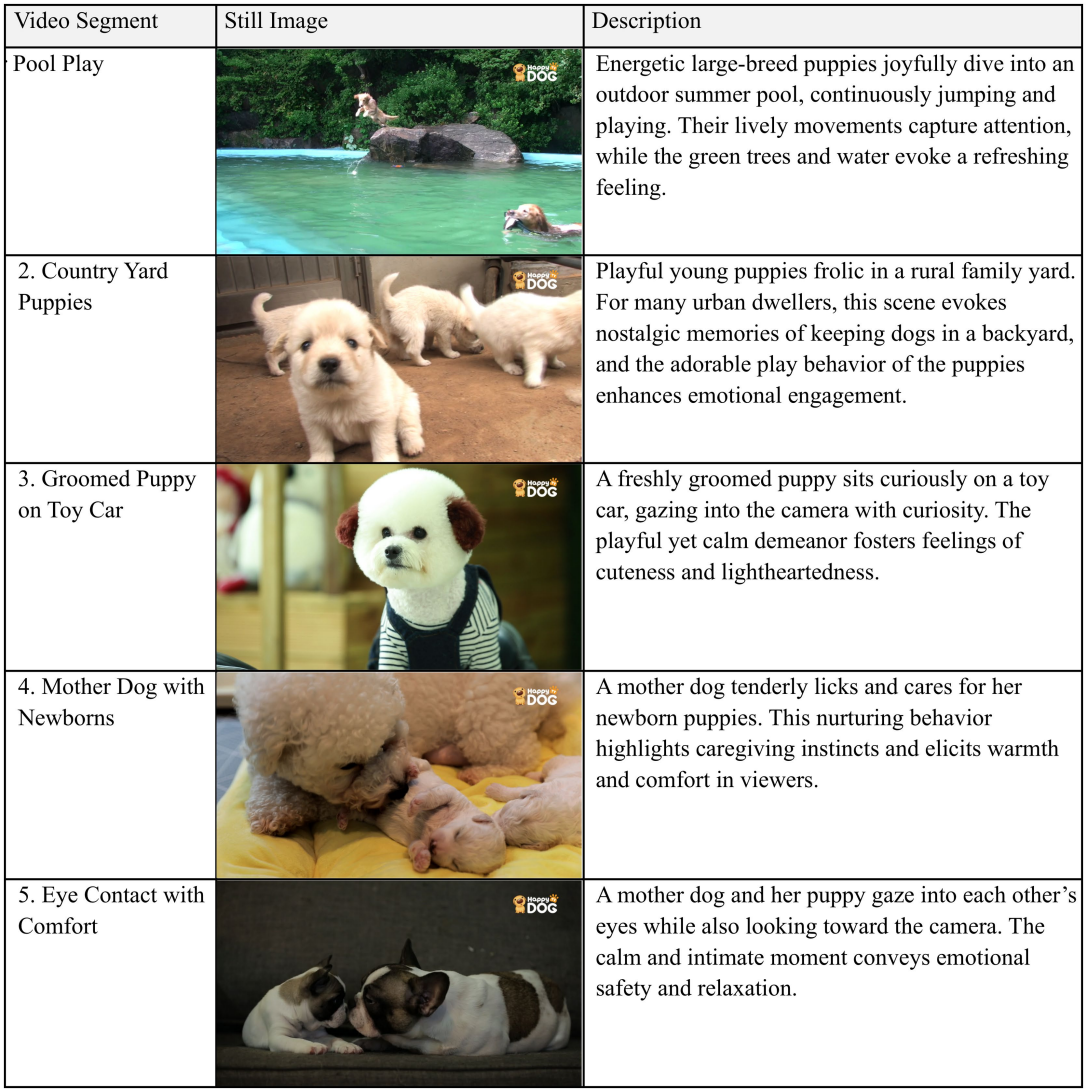
Still images and descriptions of the five video segments used in the intervention.

All participants viewed five professionally produced puppy video program (3 min each; total duration: 15 min) in quiet, sound-isolated company meeting rooms reserved for the experiment. The sessions were conducted under the supervision of the primary researcher and an assistant to ensure consistency and minimize external distractions. The videos were originally filmed in 4 K ultra-high-definition resolution to vividly capture details such as the puppies’ fur texture, facial expressions, and natural movements. Special emphasis was placed on capturing moments where the puppies gazed directly into the camera lens, creating a perceived eye-contact effect to simulate a face-to-face interactions with the viewer. This setup was intended to evaluate the potential of digital content as a visual surrogate for live animals. To enhance viewer attention and reduce interference from ambient noise, a customized audio sound designed to complement the visual content was included. The audio served only as a low-level background to support attention and was not intended as an independent therapeutic component. The volume was deliberately minimized to avoid interference with visual engagement, in line with the study’s emphasis on visual stimuli (Figure 1 and Supplementary Video S1).

### Procedure

**Figure 2:**
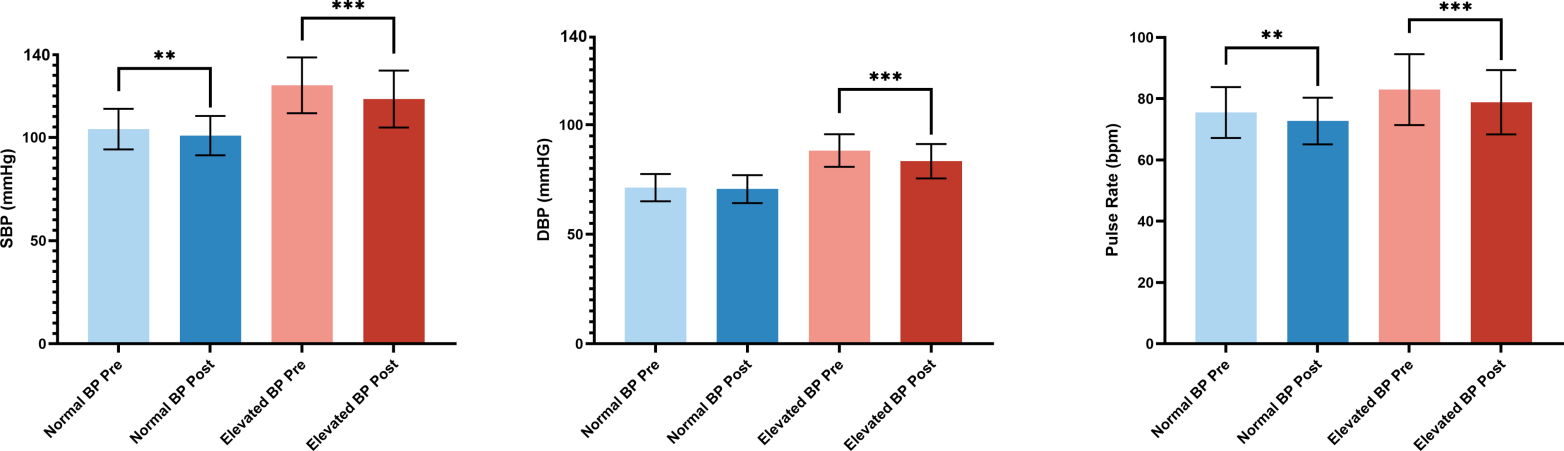
Within-group changes in physiological measures. Systolic blood pressure (SBP, mmHg), diastolic blood pressure (DBP, mmHg), and pulse rate (bpm) before (Pre) and after (Post) the intervention in the normal BP and elevated BP groups. Bars represent means; error bars represent standard deviation. **p < 0.01, ***p < 0.001; ns, not significant.

The experimental procedure was conducted in a fixed sequence. Company meeting rooms were reserved for the sessions. To minimize the influence of potential confounding factors, participants obtained sufficient sleep and abstained from caffeine, alcohol or medication for 24 h. Additionally, they were required to refrain from heavy meals for at least 2 h, and water for at least 1 h prior to the experiment. All participants rested sufficiently for 1 h prior to the commencement of the experiment. Following the stabilization period, participants completed a brief demographic questionnaire (age, sex, education, years of work, dog affinity etc.), followed by basedline psychological assessments using the STAI-S and the K-POMS-B. Next, baseline physiological measurements (SBP, DBP and pulse rate) were obtained three times consecutively using an automated device; the first reading was excluded, and the mean of the second and third readings was used for analysis. Participants then viewed the 15-min puppy video programs. After viewing the video, physiological measurements were again collected three times in the same manner, and the mean of the second and third readings was used. Finally, participants completed the same psychological assessments (STAI-S and K-POMS-B) as post-intervention measures (Figure 2).

### Psychological measures

BP and pulse rate were measured by trained personnel using a validated automated oscillometric device (Omron HEM-7156 Omron Healthcare Co., Ltd., Japan), adhering strictly to the international standard recommended procedures outlined in the 2025 AHA/ACC guidelines (31). Briefly, the assessment was performed using the correct cuff size fitted directly on the participant’s bare arm, with the arm carefully supported at heart level. Prior to the initiation of the measurements, participants were instructed to relax and sit quietly in a chair with their fell flat on the floor, leg uncrossed, and their back fully supported for a rest period exceeding 5 min. To eliminate potential external psychophysiological reactivity, talking was strictly prohibited for both the participants and the evaluators during both the rest and active measurement intervals, and any smartphone utilization was entirely banned (31).

### Psychological measures

The State–Trait Anxiety Inventory developed by Spielberger, measures anxiety through two subscales: state and trait anxiety (32). Factor analytic studies have confirmed the construct validity of the state anxiety subscale across multiple cultural contexts. This study used the 20 items assessing state anxiety (STAI-S) to capture transient stress levels. Each item was rated on a 4-point Likert scale (1- not at all to 4 = very much), with higher scores reflecting greater anxiety. The Korean version, adapted by Kim and Shin (33), is a validated instrument for which factor analytic studies have confirmed the construct validity with a reported Cronbach’s ***α*** *= 0.89.* In the present study, internal consistency was Cronbach’s ***α*** = 0.886 (pre-test) and 0.875 (post-test).

The Korean version of the Profile of Mood States-Brief is a validated, simplified, culturally adapted version of the original POMS, developed by McNair et al. (34) and revised by Yeun and Shin-Park (35). Yeun and Shin-Park (35) verified the construct validity of the Korean version through cross-cultural analysis, confirming its six-factor structure. It consists of 30 items measuring six mood states: tension, depression, anger, fatigue, confusion, and vigor. Items were scored on a 5-point Likert scale (0 = not at all to 4 = extremely). The Total Mood Disturbance (TMD) score was calculated by summing the five negative mood subscales and subtracting the vigor subscale. The Korean version was reported as Cronbach’s *α = 0.85.* In the present study, internal consistency was Cronbach’s ***α*** = 0.916 (pre-test) and *α* = 0.910 (post-test).

### Statistical analysis

All statistical analyses were conducted using SPSS Statistics 27 (IBM Corp).

We compared demographics, anthropometrics, occupational, pet-related characteristics, cardiovascular measures, and psychological measures between the normal and elevated BP groups using independent t-tests for continuous variables and chi-square tests for categorical variables.

We assessed the effects of the video viewing on the physiological and psychological parameters in the normal and elevated BP groups, using the paired t-test.

We compared these effects between the normal and elevated BP groups, controlled for confounding factors including demographics, anthropometrics, occupational characteristics and pet-related characteristics using a series of univariate analyses of covariance (ANCOVAs).

Statistical significance was established at *p* < 0.05, and the Bonferroni adjustment was applied to correct for multiple comparisons.

## Results

### Baseline characteristics of participants

Table 1 presents the demographic and baseline clinical characteristics of the enrolled office workers (36 in the normal BP group and 30 in the elevated BP group). First, there were no statistically significant differences between the normal and elevated BP groups regarding age, height, education level, years of service, pet ownership, dog affinity and all baseline psychological measures.

In contrast, the elevated BP group exhibited significantly higher values for weight, body mass index (BMI), rate of anti-hypertension medication use, and baseline BP and pulse rate. Conversely, the proportion of participants working in revenue-generating roles was significantly lower in the elevated BP group compared to the normal BP group (Table 1).

### Effects of viewing structured puppy video

#### Physiological measures

In the normal BP group, SBP and pulse rate decreased significantly whereas no statistically significant change was observed in DBP. In contrast, the elevated BP group demonstrated significant reductions across all cardiovascular parameters including SBP, DBP, and pulse rate (See Tables 2 and Figures 2A–C).

**Table 2:** Within-group changes in physiological measures (paired t-tests).

Measure	Group	Pre (Mean ± SD)	Post (Mean ± SD)	MD	*t*	*p*-value	Cohen’s *d*
SBP (mmHg)							
	Normal BP	104.03 ± 9.83	100.87 ± 9.52	3.16	3.409	0.002	0.568
	Elevated BP	125.23 ± 13.53	118.58 ± 13.78	6.65	6.256	< 0.001	1.142
DBP (mmHg)							
	Normal BP	71.29 ± 6.24	70.65 ± 6.39	0.64	0.717	0.478	0.119
	Elevated BP	88.28 ± 7.44	83.38 ± 7.84	4.93	5.184	< 0.001	0.947
Pulse rate (bpm)							
	Normal BP	75.49 ± 8.32	72.72 ± 7.63	2.76	3.600	0.001	0.602
	Elevated BP	82.95 ± 11.59	78.83 ± 10.51	4.12	4.171	< 0.001	0.762

Values are presented as Mean ± SD. MD, mean difference (Pre−Post); BP, blood pressure; SBP, systolic blood pressure; DBP, diastolic blood pressure.

#### Psychological measures

STAI-S scores decreased significantly in the normal and elevated BP groups.

Regarding the K-POMS-B mood subscales, significant reductions were observed in the normal BP group across all negative mood subscales: tension, depression, anger, fatigue, and confusion. Vigor also showed a statistically significant decrease.

**Figure 3:**
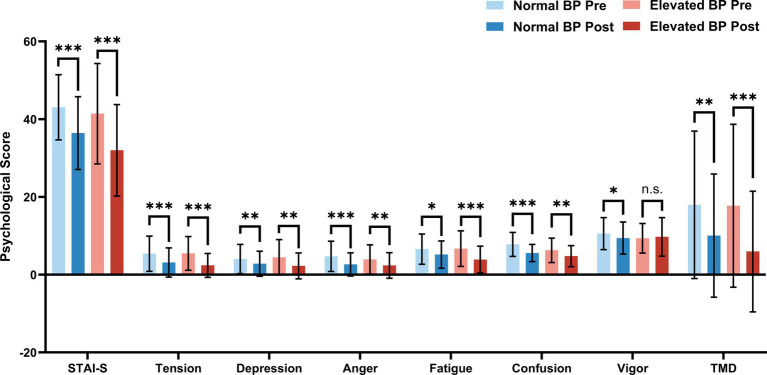
Within-group changes in psychological measures. State anxiety (STAI-S), K-POMS-B subscales (Tension, Depression, Anger, Fatigue, Confusion, Vigor), and Total Mood Disturbance (TMD) before (Pre) and after (Post) the intervention in the normal BP and elevated BP groups. Bars represent means; error bars represent standard deviation. *p < 0.05, *p < 0.01, ***p < 0.001; ns, not significant.

Similarly, the elevated BP group showed significant reductions in tension, depression, anger, fatigue, and confusion, whereas vigor showed no significant change. TMD scores decreased significantly in both groups (Table 3 and Figure 3).

**Table 3:** Within-group changes in psychological measures (paired t-tests).

Measure	Group	Pre (Mean ± SD)	Post (Mean ± SD)	MD	*t*	*p*-value	Cohen’s *d*
STAI-S							
	Normal BP	43.06 ± 8.38	36.41 ± 9.36	6.64	5.199	<0.001	0.867
	Elevated BP	41.43 ± 12.91	32.00 ± 11.75	9.43	5.671	<0.001	1.035
Tension							
	Normal BP	5.39 ± 4.53	3.14 ± 3.76	2.25	4.684	<0.001	0.781
	Elevated BP	5.47 ± 4.34	2.40 ± 3.07	3.07	6.671	<0.001	1.218
Depression							
	Normal BP	4.03 ± 3.75	2.81 ± 3.22	1.22	2.847	0.007	0.474
	Elevated BP	4.47 ± 4.57	2.27 ± 3.32	2.20	3.475	0.002	0.634
Anger							
	Normal BP	4.72 ± 3.88	2.64 ± 2.99	2.08	4.652	<0.001	0.775
	Elevated BP	3.93 ± 3.76	2.37 ± 3.30	1.57	3.781	0.001	0.690
Fatigue							
	Normal BP	6.58 ± 3.89	5.17 ± 3.53	1.42	2.544	0.016	0.424
	Elevated BP	6.70 ± 4.58	3.90 ± 3.43	2.80	5.662	<0.001	1.034
Confusion							
	Normal BP	7.78 ± 3.09	5.58 ± 2.22	2.19	4.357	<0.001	0.726
	Elevated BP	6.27 ± 3.13	4.77 ± 2.70	1.50	3.266	0.003	0.590
Vigor							
	Normal BP	10.58 ± 4.12	9.42 ± 4.13	1.17	2.263	0.030	0.377
	Elevated BP	9.37 ± 3.82	9.73 ± 4.95	−0.37	−0.580	0.564	−0.107
TMD							
	Normal BP	17.97 ± 18.97	10.06 ± 15.83	7.92	3.588	0.001	0.598
	Elevated BP	17.73 ± 20.95	5.97 ± 15.52	11.77	5.847	<0.001	1.068

Values are presented as Mean ± SD. MD, mean difference (Pre−Post); BP, blood pressure; STAI-S, State–Trait Anxiety Inventory-State; K-POMS-B subscales (Tension, Depression, Anger, Fatigue, Confusion, and Vigor). Total Mood Disturbance (TMD), Vigor is scored positively; a negative MD indicates improvement.

### Comparison of video viewing effects between normal and elevated BP groups

When comparing the physiological and psychological effects between the two BP groups after controlling for confounding factors (sex, age, height, weight, BMI, education, functional role, pet ownership, dog affinity etc.), SBP and DBP showed greater reductions in the elevated BP group. No significant differences were observed between the normal and elevated groups for remaining parameters.

## Discussion

**Figure 4:**
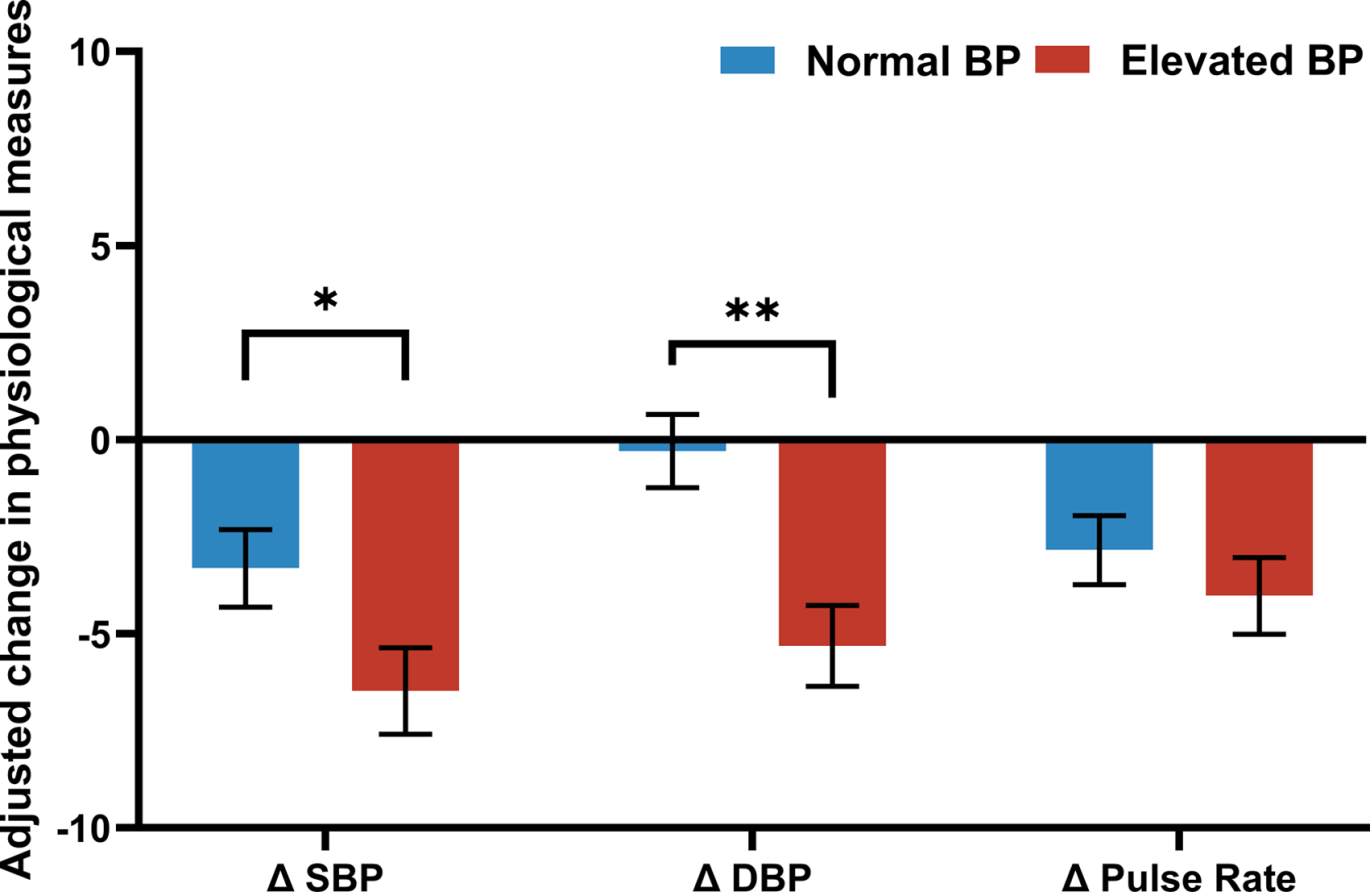
Between-group comparison of physiological measures. Adjusted mean changes (Δ = Post − Pre) in systolic blood pressure (ΔSBP, mmHg), diastolic blood pressure (ΔDBP, mmHg), and pulse rate (ΔPR, bpm) in the normal BP and elevated BP groups. Values are estimated marginal means adjusted for sex, age, functional role, and pet ownership; error bars represent standard error. *p < 0.05, *p < 0.01.

**Figure 5:**
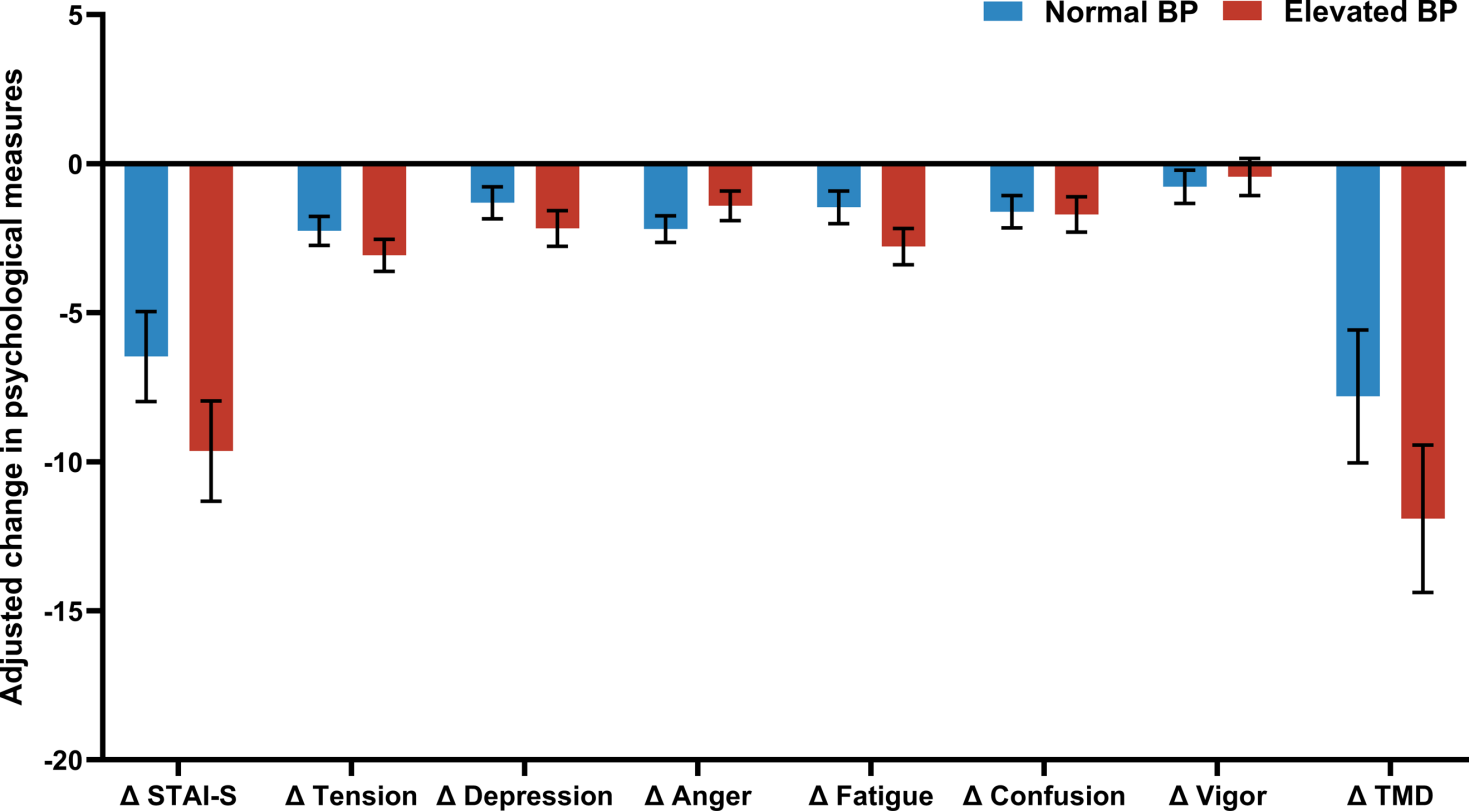
Between-group comparison of psychological measures. Adjusted mean changes (Δ = Post − Pre) in state anxiety (ΔSTAI-S), K-POMS-B subscales (ΔTension, ΔDepression, ΔAnger, ΔFatigue, ΔConfusion, ΔVigor), and Total Mood Disturbance (ΔTMD) in the normal BP and elevated BP groups. Values are estimated marginal means adjusted for sex, age, functional role, and pet ownership; error bars represent standard error. No between-group differences were statistically significant.

Although medication is the standard approach for managing high BP and stress, there is a growing interest in non-drug alternatives that can lower cardiovascular risks (Table 4 and Figures 4–5) (36). Traditional methods like meditation and yoga effectively calm the nervous system, but they require long-term commitment, expert guidance, and a significant investment of time and money (36). These requirements may pose practical barriers for contemporary office workers with high occupational demands, highlighting the need for accessible, non-invasive, digitally delivered interventions—such as structured video content—that can easily be incorporated into daily workplace settings.

**Table 4:** Comparison of adjusted changes in physiological and psychological measures between groups (ANCOVA).

Variable (Δ = Post−Pre)	Normal BP (*n* = 36) Mean ± SE	Elevated BP (*n* = 30) Mean ± SE	*F*	*p*-value	Partial η^2^
Physiological measures					
Systolic BP (mmHg)	−3.31 ± 1.00	−6.47 ± 1.11	3.897	0.049	0.061
Diastolic BP (mmHg)	−0.29 ± 0.94	−5.31 ± 1.04	11.239	0.001	0.158
Pulse Rate (bpm)	−2.84 ± 0.89	−4.02 ± 0.99	0.696	0.408	0.011
Psychological measures					
STAI-S	−6.47 ± 1.51	−9.64 ± 1.68	1.726	0.194	0.028
Tension	−2.25 ± 0.49	−3.07 ± 0.54	1.108	0.297	0.018
Depression	−1.31 ± 0.54	−2.17 ± 0.60	0.981	0.326	0.016
Anger	−2.19 ± 0.45	−1.41 ± 0.50	1.167	0.284	0.019
Fatigue	−1.46 ± 0.55	−2.78 ± 0.61	2.238	0.140	0.036
Confusion	−1.61 ± 0.54	−1.70 ± 0.59	0.013	0.911	0.000
Vigor	−0.77 ± 0.56	−0.44 ± 0.62	0.142	0.707	0.002
TMD	−7.80 ± 2.23	−11.91 ± 2.47	1.337	0.252	0.022

Values represent estimated marginal means ± standard error (SE), adjusted for sex, age, functional role, and pet ownership. Change scores (Δ) were calculated as post-intervention minus pre-intervention values. Homogeneity of error variances was confirmed by Levene’s test for all variables (all *p* > 0.05). Bonferroni correction was applied to pairwise comparisons. BP, blood pressure; STAI-S, State–Trait Anxiety Inventory-State; TMD, Total Mood Disturbance.

Several prior studies have explored how animal videos affect stress. Wells compared five silent video conditions: fish, birds, primates, a human drama, and a blank screen. The study found that pure visual stimulation can lower physical stress, showing that the three animal videos were significantly better at buffering heart rate and BP responses than the human drama or blank screen (37). Later, Ein et al. (25) compared videos of active dogs, calm dogs, nature, and a blank screen. They reported that dog videos were much better than nature or control conditions at reducing psychological anxiety and improving mood, though they found no significant changes in physical measures like BP. Similarly, Lavan et al. (38) compared videos of puppies, adult dogs, nature, and a blank screen. Their results showed that puppy videos made viewers feel significantly happier than nature or blank screens, and both dog and nature videos helped participants relax; however, physical stress signs were not evaluated for most participants. These studies, which analyzed the effects of videos, showed the potential of digital animal-assisted approaches. However, they were largely limited to university students in controlled laboratory settings. Furthermore, previous research used simple video recordings without considering cinematographic elements—such as camera angles, gaze induction, and field-of-view design—which help guide a viewer’s visual attention. To address this gap, the present study focuses on the importance of scientifically structuring visual stimuli.

The theoretical basis for developing our structured video stimuli is grounded in baby schema research. Borgi and Cirulli (27) reported that recognition of and preference for infantile physical features in animals emerge from early stages of human development. Additionally, Borgi et al. (39) used eye-tracking technology and found that faces with higher baby schema scores elicited significantly longer gaze fixation times, and that puppies received significantly higher cuteness ratings than cats and human adults in evaluations of human and animal faces. Furthermore, the eye regions of dog and cat faces were reported to attract significantly higher fixation proportions than human faces. Based on these theoretical foundations, the present study directly applied this knowledge to content design, producing structured puppy video content with precisely designed cinematography and direction in collaboration with media production professionals.

Our findings showed that following the video viewing, participants exhibited reductions in both BP and pulse rate. Concurrently, assessments of psychological parameters revealed a clear decrease in anxiety and negative mood states. Chronic negative affect and perceived loss of environmental control have been reported as key mechanisms underlying hypertension and cardiovascular risk (40). This relationship suggests that digital animal-assisted approaches may contribute to modulating stress-related physiological responses. The observed changes may be linked to the neurobiological mechanisms of the “baby schema” effect (27). Infantile physical features of puppies, such as large eyes and round faces, capture visual attention and increase gaze duration (39). Previous research suggests that these stimuli activate the neural reward network, specifically the dopaminergic midbrain and nucleus accumbens (28, 41), which can promote oxytocin release (16). Oxytocin acts as a neuromodulator that attenuates amygdala reactivity, thereby inhibiting the hypothalamic–pituitary–adrenal (HPA) axis and reducing cortisol secretion (16). This neuroendocrine cascade facilitates a shift in the autonomic nervous system toward parasympathetic dominance (16), which may account for the reductions in BP, pulse rate, anxiety, and negative mood states observed in this study.

To our knowledge, prior research has not concurrently evaluated both BP and psychological responses to structured videos among office workers in their workplace. While research related to digital therapeutics often focuses on system platforms, app infrastructure, or hardware, the role of the structured content itself remains underexplored. Future longitudinal research could explore BPV, as increased year-to-year BPV predicts future hypertension even in normotensive individuals (14). Furthermore, integrating this visual intervention with AI-based healthcare technology could support real-time biosignal monitoring and the automated delivery of personalized content (42). In conclusion, these preliminary findings suggest that structured digital content has the potential to assist in modulating acute physiological responses and supporting mood states among office workers.

### Limitations

This was an exploratory study conducted in a real-world workplace setting without a control group and with a single-session design. The absence of a control group limits the ability to attribute observed changes solely to the video intervention, and the possibility of confounding factors such as the Hawthorne effect cannot be ruled out. Additionally, subgroup classification into normal and elevated BP was based on BP measurements taken at the time of assessment, rather than established clinical criteria, which limits the interpretation of between-group differences. Future randomized controlled trials with longitudinal design and clinically defined participant groups are warranted to confirm these findings.

The inclusion of background audio may have acted as a confounding factor. Thus, the findings represent an integrated audiovisual experience rather than visual stimuli alone. Future research should test the stimuli separately to isolate their effect.

The sample in this study was limited to Korean office workers, which may restrict the generalizability of the findings to other cultures or occupational sectors. Future research involving more diverse populations is required to confirm the universal applicability of these results.

## Conclusion

This study quantitatively provided preliminary evidence that watching scientifically structured puppy videos can significantly reduce physiological and psychological stress responses in both normal BP and elevated BP adults. The intervention stimuli were engineered based on congnitive and evolutionary theories to systematically elicit positive affect, suggesting potential stress-buffering effects within within real-world corporate environments. While a substantial portion of digital health interventions emphasizes passive monitoring and biometric measurement, this study utilized digital media content itself as an active, non-pharmacological emotional intervention. These findings highlight the clinical and practical potential of content-centered digital emotional interventions to foster positive affect and elicit beneficial physiological responses, suggesting a novel pathway for scalable, accessible adjuncts to existing stress and cardiovascular health promotion stratgeies. Future research should systematically examine how design and composition methods affect outcoms across other emotionally engaging stimuli, such as other cute animals or alternative visual content, while also evaluating long-term effects and considering the incorporation of 24-h ambulatory blood pressure monitoring (ABPM) to enhance clinical relevance. Integrating such interventions with AI-driven personalized content delivery could further enhance their practical utility.

## Data Availability

The datasets are not publicly available due to privacy restrictions but are available from the corresponding author on reasonable request.

## Supplementary material

Supplementary Video S1 A short representative excerpt of the structured puppy video program used as the intervention stimulus. The full program comprised five professionally produced segments (15 min total); this excerpt presents representative scenes from those segments.
